# Pervasive Refusal Syndrome: Three Case Reports—Autism as a Predisposing Factor and Gentle Coercion to Shorten Duration of Disorder?

**DOI:** 10.1155/2022/2258180

**Published:** 2022-03-23

**Authors:** Håkan Jarbin, Ann-Sofie Saldeen, Carl-Magnus Forslund

**Affiliations:** ^1^Lund University, Faculty of Medicine, Department of Clinical Sciences Lund, Child and Adolescent Psychiatry, Lund, Sweden; ^2^Child and Adolescent Psychiatry, Region Halland, Sweden; ^3^The Research Unit, Child and Adolescent Mental Health Unit, Psychiatry Region Capital, Hellerup, Denmark

## Abstract

**Background:**

Pervasive refusal syndrome (PRS) is a severe child psychiatric syndrome not yet included in the international classification and mostly affecting girls aged 7-15 years. Hospital admission and severe loss of function extend for many months and years but most recover. Autism has been suggested as a predisposing factor but largely lacks support for typical cases of PRS. Treatment is not evidence-based and described as requiring a lengthy inpatient stay with a very gradual and sensitive rehabilitation program. *Case Presentations*. Three cases of pervasive refusal syndrome (PRS) in girls aged 9–16 years are presented to report autism as a predisposing factor and to discuss gentle coercion as part of the management strategy to speed up the lengthy recovery. The cases, which met the proposed criteria and typical background characteristics, were noted with the addition of undiagnosed autism in two cases. The duration of inpatient admission was 8–14 months. Disease duration was 15-36 months. An adequate but negative lorazepam trial to rule out catatonia was carried out. Treatment was in one case successfully expedited with gentle coercion within a transparent management plan. Rehabilitation was slower in PRS with comorbid autism; additionally, accommodations to school and living support needed to be put in place.

**Conclusions:**

PRS is a useful clinical entity and best perceived as a primitive reaction to overwhelming stress rather than as catatonia. Autism might be another predisposing factor and needs to be assessed. A psychoeducational approach and a clear management plan support rehabilitation. A gentle coercion might hasten recovery.

## 1. Background

Pervasive refusal syndrome (PRS) is a rare but severe and clinically challenging condition mostly afflicting girls aged 7-15 years. The core symptoms contain regression in behaviour with inability to eat, talk, move, and self-care along with an angry refusal of help. It was first described in four patients by Lask in 1991 [1] while reviews by Jaspers et al. in 2009 found 24 cases [2] and by Otasowie et al. in 2020 found 38 cases excluding 41 cases of PRS in asylum seekers [3]. The course is protracted, but most patients are reported to recover [2, 3]. Criteria were first introduced by Thompson and Nunn in 1997 [4] and adapted by Jaspers et al. in 2009 [2] (Table 1). Cases are reported from Europe and Australia as well as one case from India, but no cases are reported from the U.S. [3]. PRS is not yet recognized in the DSM or ICD nomenclature [5, 6]. The aetiology was initially suggested to be traumatic related to sexual abuse [1]. Later, the concept of learned helplessness was put forward. Perceived uncontrollability and resignation from life challenges were assumed to be the core driving force in PRS [7]. A neurobiological explanation was proposed integrating the core features: refusal driven by sympathetic hyperarousal and behaviour paralysis driven by parasympathetic hyperarousal [8]. A phenomenological model integrating the common findings proposed a range of predisposing factors: (1) sensitive personality, (2) previous psychiatric issues, (3) parental psychiatric problems, and (4) stressful life events [9]. Undiagnosed autism was recently suggested as another vulnerability factor [3, 10]. There has also been suggestion of PRS as a form of catatonia [11] but not validated by any case of PRS successfully treated with benzodiazepines or electroconvulsive treatment (ECT) in line with a diagnosis of catatonia [3].

PRS overlaps with a range of psychiatric disorders such as depression, selective mutism, anxiety disorders, chronic fatigue syndrome, anorexia nervosa, somatisation disorder, conversion disorder, factitious disorder, and catatonia [2, 3]. However, none of these diagnoses accounts for the specific constellation of symptoms with pervasive refusal but without other features of anorexia nervosa, depressive or schizophrenic disorder, and severe regression without help-seeking behaviour or secondary gain from illness behaviour, which could be a driving force in asylum seekers [2, 3].

Treatment with a multidisciplinary approach and a structured management plan was first proposed by Nunn et al. from experiences with seven cases [12]. To avoid enmeshment, parents were separated from the patient with strict rules for visits and phone contacts while staff provided the only care. Threats, punishment, praise, and incentives were strictly avoided based on previous learning, while gentle coercion and clear communication to the patient about steps in treatment were suggested. Outcome was generally good, but costs were substantial with admissions in the 1-2 year range and the lurking risk of staff burnout leading to a punitive approach [12]. Jaspers et al. also emphasised a long-term management plan, involving the family in the planning while creating some distance and avoiding pressuring the patients but promoting activities just on demand as opposed to gentle coercion [2]. Otasowie et al. highlighted patients' perception of care as possibly coercive and the subsequent risk for compassion fatigue and burnout in staff [3]. Medications are generally deemed ineffective except for comorbidities [2], while a vigorous trial of benzodiazepines and/or ECT is advocated by U.S. clinicians [11].

We present three recent Swedish cases of PRS in nonasylum seekers to expand on autism as another vulnerability factor and to discuss the potential of active and gentle coercive rehabilitation to speed up recovery.

## 2. Ethics

Ethical approval of reported cases has been obtained from the Swedish Ethical Review Authority (No. 2021-00216). Patients and parents have read the manuscript and consented in writing. The case report guidelines (CARE) checklist for clinical case reports [13] is adhered to and reported in supplemental table 1.

## 3. Case 1

**Figure 1:**
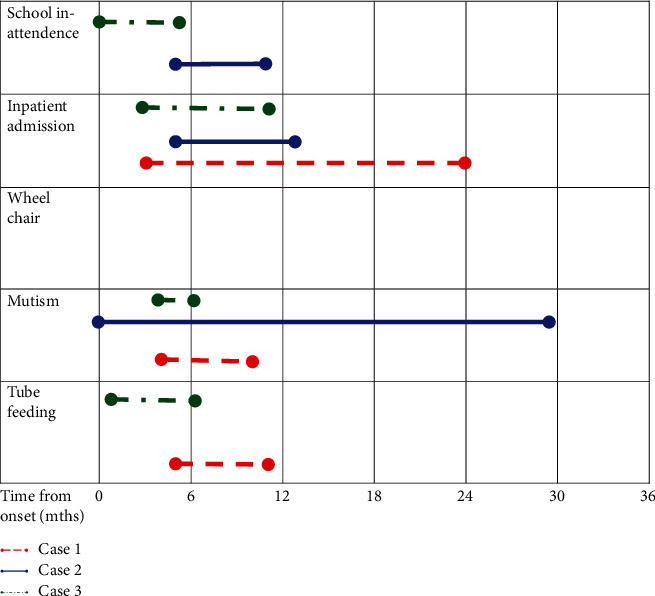
Timeline from onset to functional changes.

This 16:7-year-old girl presented at the emergency department with inability to walk and mostly mute after a few months of increasing fatigue and loss of appetite. She was described as a cautious, conscientious, and withdrawn child. She was independent from an early age and very rarely asked for support or care. Idiosyncratic language was also described. The family was intact but strained for several years as her father suffered from an undiagnosed neurological condition causing her symptoms of posttraumatic stress. She was ambitious at school and with extreme auditory memory. At the start of high school, she skipped the first year. She soon contacted the school psychologist due to mild depressive symptoms and a feeling of being different. She performed excellently on a WISC test of intelligence. Autism was suspected, but the family declined a referral to Child and Adolescent Mental Health Service (CAMHS). From January onward, fatigue, reduced appetite, and social isolation progressed. In March, she was assessed at CAMHS emergency department with normal neurological status and blood chemistry. Assuming depression, fluoxetine was started. In April, she stopped walking, was mainly mute, and had to be fed by her mother. She was this time admitted to the CAMHS inpatient unit. An extensive workup ruled out organic causes. Bush Francis Catatonia Rating Scale (BFCRS) was applied, but a total of 5 points which was not indicative of catatonia. PRS was suggested as a working diagnosis after 3 weeks of inpatient care. Treatment as suggested by Jaspers et al. [2] was started including daily physiotherapy with a care dog and sessions with psychiatrist and psychologist. At this point, she communicated with tapping fingers for yes and no and displayed violent outbursts and screaming when attempts were made to make her talk or move. She could, via her mother, formulate different statements and questions, which were elaborated on in the supportive sessions. She was presented the neurobiological model of PRS [8] finding it very appropriate for her condition. She felt that “everything was a threat.” She became, after two months of admission, totally mute and refused to eat and drink but accepted nasogastric tube feeding. Next month, a zolpidem diagnostic test for catatonia was carried out, but BFCRS at 7 before and at 6 after the test was inconclusive. A lorazepam trial of 13 days and up to 8 mg was unsuccessful and discontinued due to sedation. Electroconvulsive therapy was not considered as catatonia score was low. Medication with fluoxetine up to 30 mg for five months was deemed ineffective and was discontinued. Attempts to a more coercive activation were made but immediately backfired resulting in deterioration and regression. A gentle sensory approach to the smell of flowers, massage, and taste of ice cream was continued, and gradually, small progress was noticed. She sang her first words after six months of silence, “I am in charge.” Gradually, she was able to describe symptoms of inattention, autism, and posttraumatic stress, which were corroborated by information from family and friends. A couple of months after her first words, she gradually resumed eating but with the support of nasogastric tube feeding along with being more communicative and allowing wheelchair outings with her mother. Tube feeding was discontinued after seven months of inpatient care. She selectively talked. Methylphenidate was successfully trialed as she expressed “inner chaos” and reported subclinical inattention. Further progress in talking and moving, including a wheelchair trip to a museum, enabled discharge to a nursing home after 14 months of inpatient treatment. At follow-up 6 months after discharge, she had improved, talking without problems and moving freely indoors, while still using the wheelchair outside. At follow-up 17 months after discharge, she still lived in a nursing home but reported full recovery from PRS from 7 months after discharge from the ward (Tables 2–4, Figure 1).

*Patient's comment:*

My PRS had caused my emotions to fluctuate and often feel overwhelming. In the hospital I was received as I am, with my autism. It was a genuine meeting, which gave me a feeling of being part of life, not being alone. Long-term trust was built. I got help with many issues, including using my imagination of a mental safe place to feel calm or going for walks was also of great value to me. Through the care, I started for the first time to address my problems.

*Parents' comment:*

A liberating utterly stress-free environment with tender loving care constituted the basis for her recovery. The patience of a coordinated and dedicated team of healthcare professionals supporting our daughter's human dignity, the search for knowledge and free will was crucial during the prolonged admission.

## 4. Case 2

This girl of 11:7 years presented at the emergency department with difficulty to both walk and speak. She exhibited typical predisposing factors regarding family history, comorbid diagnoses, precipitating stress, and virus infection. Her mother had suffered from depressions. She had autistic traits, increasing social issues with peers, and difficulty expressing herself. She was unhappy going to school for several years due to issues with girls at school. The girl was close to her mother and found her parents' divorce very difficult, this was at age 11:1 years, 6 months before her breakdown. A couple of months after the parents moved apart, she took on the responsibility of caring for her depressed mother. Later, she went on a trip abroad with her father and was at the time already developing some difficulty walking. After returning, she contracted a throat infection one month before onset of PRS. Even though the fever wore off, she did not get well. She had fatigue and pain in her legs and could not walk, as well as becoming increasingly mute. Her parents took her to several doctors, and she was unsuccessfully treated with several antibiotics.

The girl was thoroughly investigated at the hospital to exclude organic aetiology. She could not walk, stopped talking, and finally closed her eyes. The girl was admitted to child psychiatric inpatient care and diagnosed with PRS according to criteria by Jaspers [2] but not the original criteria by Nunn [7] as she never refused food.

Her parents had a hard time to understand the disease and did not truly trust the psychiatrists on the diagnosis of PRS. They were sometimes impatiently trying to make her look or walk, which brought angry reactions. During her hospital stay, she attended school, sessions with a psychologist with whom she communicated in writing with a psychiatrist and a physiotherapist. She much appreciated the dog of the physiotherapist. She was at the hospital unsuccessfully treated with sertraline 150 mg daily for 8 months. In sessions with a psychologist, she repeatedly brought up difficulties with her parents pressing her to do things she was not able to. She went home on leave all weekends. She could enjoy her time on her own, talking to herself, walking around, and opening her eyes.

A very gentle rehab process was started based on her ability to move when not seen, but progress was limited and slow. The parents and staff never confronted her with her ability to walk, talk, and use her eyes when being alone. During the admission, she started using arms and hands and more expressive in gestures and writing. She seemed less depressed. There were a few unsuccessful trials to coerce her into activity. However, towards the end of the hospital stay, her parents accepted the diagnosis and were more understanding of her symptoms avoiding to press her. They also moved together. At the end of the hospital stay, she began to use sunglasses and was able to open her eyes. She gained about 20 kg weight during her stay due to sertraline but also to her immobility. At discharge after 8.5 months, she still needed a wheelchair and was mostly mute.

After discharge, she spent almost a year at home with very little progress. At that point, her parents met with the parents of case 3 and got inspired to try more active measures. They decided to take away the wheelchair, and shortly after, she was able to walk. Some months later, she got contact with a speech therapist and gradually started to talk. Today she goes to a special school with small classes and has few social contacts. However, she has recovered from PRS. She walks and talks without problems and her parents believe that they have fully got their daughter back again (Tables 2–4, Figure 1).

*Comments from the patient:*

I wished you had a speech therapist during my hospital stay. I don't think I was ready for challenges when I was at the hospital.*Comments from parents:*

We wished we had met parents with PRS experience at an early stage so we could learn from them and trust the treatment that was given at the hospital. We would have preferred the ward to be more active in pushing our daughter to activities. It was very useful for us to meet the parents of another girl with PRS (case 3) from another hospital but with shockingly similar symptoms. We could better trust the diagnosis of PRS but also be hopeful and stronger as the other patient had recovered. Information in writing or other parents to talk with in the beginning of the disease would have been helpful.

## 5. Case 3

This girl of 9:8 years presented to the paediatric emergency department after a rapid loss of weight and increased fatigue. She had suffered from nausea and vomiting since two months extending to a vomiting phobia and rapidly decreasing food intake and weight. CBT sessions had been ineffective, and her rapidly deteriorating state required admission to the paediatric ward for tube feeding. An extensive workup ruled out organic causes. She was transferred to the child psychiatric ward as the condition was deemed functional.

The girl had always had a big heart for others and was conscientious and fearful of making mistakes. There was a family history of anxiety disorders, obsessive compulsive disorder (OCD), and depression. She had developed normally, enjoyed friends, school, and horseback riding, but suffered from a mild generalized anxiety disorder (GAD) from age 8 years. She had a close relation with her mum including also comforting her mum at times. During the half year before onset, she was slightly uneasy with leaving the house on her own in the morning and with a brief period of marital discord. She suffered several episodes of infections such as flu, gastroenteritis, and common cold. At school, she twice suffered head trauma in physical education followed by nausea and vomiting.

A diagnosis of PRS was given at an early stage at the child psychiatric ward. Her dad praised her, during the intake procedure, for being helpful while moving her from the wheelchair to a chair. She reacted intensively and immediately refrained from being helpful, thus exhibiting the typical symptom of refusal and clearly as opposed to her otherwise forthcoming personality. PRS was based on her distinct refusal, severe regression without any prominent symptoms of depression, psychosis, catatonia, or eating disorder. She was in a wheelchair, spoke quietly and minimally, and walked inside on her own. Within weeks, she stopped talking altogether and was confined to wheelchair. Sertraline 50 mg was started for GAD along with quetiapine 25 mg for sleep.

Her parents were during the first week informed about the working diagnosis of PRS and the hypothesis of overwhelming stress and helplessness [8]. To avoid parental burnout and overinvolvement, we started a weekly schedule with alternating care. Weekends, she had three days leave at home, then one day at the ward with a parent, three days at the ward by herself, and lastly another half day at the ward with a parent before next leave for home. This alternate 50/50% schedule enabled her parents to keep up their ordinary work and daily routines while also being involved in her care, while she much preferred being on leave to her room at home. Furthermore, stimulation from her usual life and extended family was continually at hand. The extended family was soon gathered to a psychoeducational session on PRS. A coerced activation was planned in liaison with parents, scheduling one hour of participation in daily life every day, which could be floating with devices in a secluded and warm pool or being pushed in a wheelchair along the beach to later have relatives or friends come to see her and later on visiting friends with a parent in spite of her refusal. New steps with biweekly slightly more socially challenging tasks were announced two weeks ahead. Every new step, no matter how minimal, triggered angry protests but was anyhow managed. Beyond one daily hour of activation, she was allowed to retreat to her room but should participate in her wheelchair with meals and snacks on the ward and at home. She reached her worst point about one month after admission: mute, whimpering and at times howling, eyes closed, needing support to stay upright in a wheelchair but vigorously and physically resisting being taken to her daily one hour floating in a secluded pool. She was tube fed at mealtime with bolus doses. After meals, she was resting in her room for an hour without any staff entering or even checking in with her to enable her to feel safe and possibly to be able to start moving on her own. We noticed but never mentioned some minor traces of her having moved about and that a few Smarties goodies were missing from a bowl in her room.

After a long summer vacation and three months of alternate care admission, she started at the hospital school one hour per day, initially with intense refusal. Furthermore, she started to have meals on her own in her room. The tube was pulled. The gently coerced activation included meeting with best friends but still confined to one hour of daily activation with biweekly increments. Six months after admission, she was ready to take the first step to her regular school, first just meeting teachers and later on participating from behind a portable wall. When school days in her hometown were extended, the leaves were increasingly longer, but the minimum two days each week was for rest on the ward, both for herself and most so for her parents. She was still strongly refusing every new step and vehemently opposing any reports of improvement. She showed her parents her anxiety and distress and later on also physical aggression to avoid further moves towards her normal life. However, when she bit by bit experienced that meeting with friends and attending school were manageable and meaningful, the resistance slowly waned after about eight months of alternate care admission and she was discharged but with continued biweekly family sessions and phone support. Six months after discharge, she was back to full time studies, commuting with the school bus and enjoying her friends and leisure activities. A year later, sertraline was slowly discontinued but subsequently restarted due to emerging symptoms of generalized anxiety and mild depression (Tables 2–4, Figure 1).

*Comments from patient:*

*Afterwards and in hindsight, I appreciate that the treatment did have elements of activation even though I really did not like this when I was ill. A helpful thing was that parents or staff never praised my small improvements and that I was allowed to start snacking and moving about in my room without any comments.*

*Comments from parents:*

*We appreciated that the psychiatrist was experienced with similar cases and that we were part of making up a detailed plan for rehabilitation with regular updates. Alternating care between home and ward was another important part to help us cope with the extended illness duration.*

## 6. Discussion

The three cases are well in line with the diagnostic criteria (Tables 1 and 3), fit also well with the typical pattern of predisposing and precipitating events (Table 2) and with complete but various time to remission (Table 4). Psychiatric assessments were performed by the CAMHS senior clinician. The diagnoses of PRS were arrived at after organic and other psychiatric diagnoses were excluded or assessed as comorbidities and criteria for PRS were fulfilled.

### 6.1. PRS as a Reaction to Overwhelming Stress

We believe in line with most authors [2, 3, 8, 15] but at odds with some [11] that PRS can be seen as a way to cope with overwhelming stress and challenges. This concept is in line with an update in psychobiological research on helplessness as a basic default mechanism when all other options from a subjective point of view seem exhausted [16]. Maier and Seligman, the original authors of the learned helplessness metaphor, rephrased helplessness from extensive neurobiological research during 50 years as an inborn default mechanism rather than a learned model [16]. All three cases experienced significant stress prior to onset. Furthermore, they later could describe a wish to avoid stress and to regain control. Case 3 clearly described spot on how she, in the prodromal phase, gave in to the temptation of somatic symptoms to avoid challenges in life. Moreover, their parents found the theory about helplessness fitting with their experience of disease onset and to be helpful in understanding the steps to health and regaining function.

### 6.2. Autism Is a Predisposing Factor

The two cases with autism or with autistic traits fit well into the concept of overwhelming stress as social challenges are greater in autism. Our cases with autism were undiagnosed at the onset of PRS. Case 1 had just changed school and jumped one year to socially more mature peers. Case 2 was overwhelmed by both parental discord and by issues with friends. In an earlier case report, a 9-year-old boy with preexisting diagnoses of autism, attention deficit disorder, and low-average IQ contracted PRS with refusal of food and fluid, of personal care, and of school and mutism. The report highlights a strong dislike of a haircut leading up to the refusal while relational stress was not elaborated but can be presumed. Moreover, he was described to respond to rules, boundaries, and consequences at odds with the standard course. Otasowie et al. discussed possible autism spectrum issues in another two case reports [3], including a boy of four years as the youngest patient diagnosed with PRS [17]. The question was raised that undiagnosed autism might be a factor in more PRS cases as autism is underdiagnosed in girls and the inability to cope with transition and catatonic features not seldom seen in autism [3]. Our cases no. 1 and 2 give support to the hypothesis that autism might be contributing, although case 2 has still not given the diagnosis but has many autistic traits. They fit well into the presumed undiagnosed autism in girls, who experience severe stress on transition in adolescence. The psychiatric evaluation in PRS needs to closely take autism into account.

### 6.3. Catatonia and Treatment with Benzodiazepines or ECT

Catatonia and its treatment with benzodiazepines and ECT have been highlighted during the last ten years and also catatonia within autism [18, 19]. Our case 1 was assessed regarding catatonia but with a low score. However, an adequate but negative benzodiazepine trial was anyhow performed. A negative lorazepam trial, i.e., nonresponse of motor symptoms to lorazepam, makes a diagnosis of catatonia unlikely but does not rule it out. A response to electroconvulsive therapy (ECT) is a more definite test of catatonia. There was an ethical dilemma to proceed with electroconvulsive treatment given the low suspicion of catatonia. The first author (HJ) had earlier been involved with another male case of PRS unsuccessfully administered three bilateral followed by three unilateral ECTs. Autism was later on diagnosed. We admit that six ECTs or just an adequate lorazepam trial is not exhaustive, but our experience does not support the catatonia hypothesis. This is in line with further case reports of PRS summarised by Otasowie et al. [3]. Further, the very typical strong reaction to praise in PRS, the ability to walk and eat if not observed, and patients' recollection of escaping into PRS are at odds with a diagnosis of catatonia. The evaluation of PRS needs to take catatonia into consideration as a differential diagnosis, but benzodiazepines or ECT is not called for in typical cases of PRS.

### 6.4. Treatment: Follow the Patient or Lead the Patient?

Our three cases underwent quite different management strategies. An early and psychoeducational approach as in cases 1 and 3 was much appreciated. Moreover, framing of the treatment as a way to overcome overwhelming stress and find the way back was helpful. A clear and agreed care plan with stepwise goal setting was much appreciated by the parents of case 3.

A common theme in management is “tender and loving care” but also to wait until the patient is ready and initiates next step [2], which was adhered to in case 1. She was supported and activated “just on demand” [2]. The initial coercive steps during admission for cases 1 and 2 were spontaneous and at times in a distraught way and not framed as part of a management plan. The coercive steps quite clearly ran into resistance from patients, while there were few preparations to deal with this resistance.

The gentle coercion was described by Nunn et al. [12] and illustrated with a similar struggle to case 3 to get the patient with PRS into the pool for hydrotherapy. Nunn et al. underline the persistent, nonpunitive, predictable, and very transparent helping process but also gentle coercion [12]. An 11-year-old girl with mild PRS was reported to respond well to “graded, sensitive, firm, and persistent approaches” regarding getting out of bed into school or ward activities [20]. The coercive strategy in case 3 was in part copied from the treatment protocol for eating disorders where a kind but firm restoration of eating sometimes has to be carried out before the child is committed but with the best interest of the child in mind. The age difference could play a part as case no. 3 with successful coercion was just 9:11 years while case 1 was 16:11 years at admission. It is well known from family-based therapy in anorexia nervosa that the model suits better at a younger age [21]. The successful coercive steps in case 3 were introduced after overwhelming stress and helplessness were elaborated on. Her suffering was validated to the patient, parents, and extended family. The coercive steps were integrated into a management plan building on the construct of PRS. We agreed with her parents that an hour of exposure to little bits of activity each day in spite of her refusal could help her to faster regain a sense of mastery and ability to manage. Each step was detailed with the parents to be just reasonably challenging and something she actually would have liked. At the start, it was strenuous to implement the hour and she had to be carried while physically resisting. However, parents and staff were convinced and expected and prepared for physical measures to enable her to float in the warm pool. As the coercive strategy turned out positive, the model with one hour of activation each day became the modus operandi. The parents of our case 2 were brought to a meeting with the parents of case 3 after the successful coercive activation to regular school and friends. After discharge, the parents of case 2 agreed on some incremental steps like taking away the wheelchair, which seemed helpful and pushed her into walking on her own. The course was, however, more protracted. From a subjective point of view, both case 1 (no coercion) and case 3 (continuous coercion) appreciated the way their rehab was designed, although case 3 was open with her dislike of the activation at the time but later believed the coercion was helpful to recover.

The possibility of reducing the disease duration and loss of life years in adolescence was driving the more active strategies. Time frames are visualized in Figure 1. The finding from Thompson and Nunn that episode duration might be dependent on the time from onset until diagnosis and treatment for PRS [4] is not supported from these few cases. Active rehabilitation with mild coercion might have speeded up the recovery time for case 3. Recent follow-up data from a naturalistic cohort of children with resignation syndrome, much similar to PRS, showed that structured activation rather than just waiting for the patient to be ready seemed effective in speeding up recovery [22]. However, great caution to aggressive coercion to reduce inpatient admissions and costs is called for. Moreover, coercive strategies might be more difficult to apply in autism as well as in older adolescents.

## 7. Strengths and Limitations

The main weakness is the anecdotal and unsystematic selection inherent in case reports. This precludes conclusions about treatment. A strength is the follow-up until remission and reflective comments from the patients and parents. The lorazepam test was also sufficient and pushed until side effects limited use. Furthermore, the conjoint meeting with parents of case two and case three along with the authors helped to carve out the similarities and differences and especially among the parents to arrive at a significant recognition of the same syndromic picture. The successful utilisation of strategies from case three by the parents of case two gives additional support to consider some coercive measures.

## 8. Conclusions

- PRS is a useful clinical entity and best perceived as a primitive reaction to overwhelming stress rather than as catatonia
- Autism might be another predisposing factor and needs to be assessed
- A psychoeducational approach and clear management plan support rehabilitation
- A gentle coercion might speed up recovery

## Figures and Tables

**Table 1:** Diagnostic criteria for PRS as adapted by Jaspers et al. [2].

1) Partial or complete refusal in three or more of the following domains: (1) eating, (2) mobilization, (3) speech, and (4) attention to personal care
2) Active and angry resistance to acts of help and encouragement
3) Social withdrawal and school refusal
4) No organic condition accounts for the severity of the degree of symptoms
5) No other psychiatric disorder could better account for the symptoms
6) The endangered state of the patient requires hospitalization

**Table 2:** Background characteristics.

	Case 1	Case 2	Case 3
Age of onset (years:months)	16:7	11:7	9:8
Gender	Female	Female	Female
Family history	Autistic traits	Depression	Obsessive compulsive disorder, depression, panic disorder
Personality	High achiever, perfectionistic, anxious, conscientious	Caring, rigid	Perfectionistic, anxious, caring, conscientious
Family setting	Intact, youngest of three sisters	Divorcing, youngest with an older brother	Intact, only child
Comorbidity	Posttraumatic stress disorder, autism, subclinical inattention	Autistic traits, subclinical inattention	Generalized anxiety disorder, panic attacks
Stressors	Undiagnosed autism, domestic brawl	Parental divorce, conflicts among peers	Parental discord, mother-child overinvolved
Precipitators	—	Throat infection	Flu, gastroenteritis, head trauma

**Table 3:** Features of inpatient stay.

	Case 1	Case 2	Case 3
Time from onset to admission (months)	3	5	3
Medical workup	LP, EEG, MRI brain, neurology examination, comprehensive blood analysis	LP, EEG, MRI brain, CT scan neck, neurology and ear, nose, and throat examination, comprehensive blood analysis	LP, EEG, neurology examination, comprehensive blood analysis
Tube feeding (months)	6	—	6
School absence (months)	Dropped out at onset	5	10
Enuresis (months)	—	—	2
Mutism (months)	6	30	2
Wheelchair (months)	18 indoors, 21 outdoors	29	4 indoors, 5 outdoors
Angry resistance	+	+	+
Activation	Gentle	Gentle (partly coercive after discharge)	Coercive
Medication	Fluoxetine, olanzapine, methylphenidate, lorazepam test	Fluoxetine, sertraline	Sertraline, quetiapine
Duration of inpatient stay (months)	14	8.5	8

LP: lumbar puncture; EEG: electroencephalography; CT: computerized tomography; MRI: magnetic resonance imaging.

**Table 4:** Follow-up.

	Case 1	Case 2	Case 3
Episode duration (months)	18	36	15
Time from admission to last follow-up (months)	18 and 31	36	62
Follow-up: clinical global impression [14]—severity of PRS (1–7)	Normal (1) but impaired by autism	Normal (1) but impaired with few social contacts	Normal (1) and generalized anxiety and depression in remission as well
Key statement	Patient's first words after 6 months of mutism: “I'm in charge.”	Patient after recovery: “I wish I could have stayed at home for treatment since being at the hospital was very stressful.”	Patient after recovery: “I was so relieved from life stresses during my episodes of somatic illness that I did not wish to get well but to dwell in seclusion.”

## Data Availability

The available data are presented in the case vignettes. Minor details have been edited to preserve confidentiality. There are no additional data.

## Abbreviations

BFCRS: Bush Francis Catatonia Rating Scale
CAMHS: Child and Adolescent Mental Health Service
CARE: Case report guidelines
CT: Computerized tomography
DSM: Diagnostic and Statistical Manual of Mental Disorders
ECT: Electroconvulsive treatment
EEG: Electroencephalography
GAD: Generalized anxiety disorder
ICD: International classification of disorders
LP: Lumbar puncture
MRI: Magnetic resonance imaging
OCD: Obsessive compulsive disorder
PRS: Pervasive refusal syndrome
PTSD: Posttraumatic stress disorder.

## Supplementary Materials

Supplementary Materials The case report guidelines (CARE) checklist for clinical case reports [13] is adhered to and reported in supplemental table 1.

## References

[1] Lask B., Britten C., Kroll L., Magagna J., Tranter M. (1991). Children with pervasive refusal. *Archives of Disease in Childhood*.

[2] Jaspers T., Hanssen G. M., van der Valk J. A., Hanekom J. H., van Well G. T., Schieveld J. N. (2009). Pervasive refusal syndrome as part of the refusal-withdrawal-regression spectrum: critical review of the literature illustrated by a case report. *European Child & Adolescent Psychiatry*.

[3] Otasowie J., Paraiso A., Bates G. Pervasive refusal syndrome: systematic review of case reports. *European Child & Adolescent Psychiatry*.

[4] Thompson S. L., Nunn K. P. (1997). The pervasive refusal syndrome: the RAHC experience. *Clinical Child Psychology and Psychiatry*.

[5] American Psychiatric Association (2013). *Diagnostic and Statistical Manual of Mental Disorders (DSM-5)*.

[6] World Health Organization (2018). *International Statistical Classification of Diseases and Health Related Problems*.

[7] Nunn K. P., Thompson S. L. (1996). The pervasive refusal syndrome: learned helplessness and hopelessness. *Clinical Child Psychology and Psychiatry*.

[8] Nunn K. P., Lask B., Owen I. (2014). Pervasive refusal syndrome (PRS) 21 years on: a re-conceptualisation and a renaming. *European Child & Adolescent Psychiatry*.

[9] Von Folsach L. L., Montgomery E. (2006). Pervasive refusal syndrome among asylum-seeking children. *Clinical Child Psychology and Psychiatry*.

[10] Bond E. C., Oliphant R. Y. K. (2018). Pervasive refusal syndrome in autistic spectrum disorder. *Case Reports in Psychiatry*.

[11] Dhossche D., Kellner C. H. (2015). Pervasive refusal syndrome: a misnomer for catatonia. *Asian Journal of Psychiatry*.

[12] Nunn K. P., Thompson S. L., Moore S. G., English M., Burke E. A., Byrne N. (1998). Managing pervasive refusal syndrome: strategies of hope. *Clinical Child Psychology and Psychiatry*.

[13] Gagnier J. J., Kienle G., Altman D. G. (2014). The CARE guidelines: consensus-based clinical case report guideline development. *Journal of Clinical Epidemiology*.

[14] Busner J., Targum S. D. (2007). The clinical global impressions scale: applying a research tool in clinical practice. *Psychiatry (Edgmont)*.

[15] Schieveld J. N. M., Sallin K. (2021). Pervasive refusal syndrome revisited: a conative disorder. *European Child & Adolescent Psychiatry*.

[16] Maier S. F., Seligman M. E. (2016). Learned helplessness at fifty: insights from neuroscience. *Psychological Review*.

[17] Taylor S., Dossetor D. R., Kilham H., Bernard E. (2000). The youngest case of pervasive refusal syndrome?. *Clinical Child Psychology and Psychiatry*.

[18] Wachtel L. E., Dhossche D. M., Kellner C. H. (2011). When is electroconvulsive therapy appropriate for children and adolescents?. *Medical Hypotheses*.

[19] Weiss M., Allan B., Greenaway M. (2012). Treatment of catatonia with electroconvulsive therapy in adolescents. *Journal of Child and Adolescent Psychopharmacology*.

[20] McGowan R., Green J. (1998). Pervasive refusal syndrome: a less severe variant with defined aetiology. *Clinical Child Psychology and Psychiatry*.

[21] Davis L. E., Attia E. (2019). Recent advances in therapies for eating disorders. *F1000Research*.

[22] Sallin K., Evers K., Jarbin H., Joelsson L., Petrovic P. (2021). Separation and not residency permit restores function in resignation syndrome: a retrospective cohort study. *European Child & Adolescent Psychiatry*.

