# Supplementary material for: Pervasive Refusal Syndrome: Three Case Reports—Autism as a Predisposing Factor and Gentle Coercion to Shorten Duration of Disorder?
Source: Case Rep Psychiatry. 2022 Mar 23;2022:2258180. doi: 10.1155/2022/2258180 (PMC8967588; doi:10.1155/2022/2258180)
Supplement: Supplementary Materials — The case report guidelines (CARE) checklist for clinical case reports [13] is adhered to and reported in supplemental table 1. [file 2258180.f1.pdf]

CARE
case report guidelines

CARE Checklist of information to include when writing a case report

(cc) BY-NC-ND

✓

Topic	Item	Checklist item description	Reported on Line
**Title**	1	The diagnosis or intervention of primary focus followed by the words "case report"	1
**Key Words**	2	2 to 5 key words that identify diagnoses or interventions in this case report, including "case report"	30
**Abstract (no references)**	3a	Introduction: What is unique about this case and what does it add to the scientific literature?	16, 20-2
	3b	Main symptoms and/or important clinical findings	18-24
	3c	The main diagnoses, therapeutic interventions, and outcomes	12, 20-1, 23-4
	3d	Conclusion—What is the main "take-away" lesson(s) from this case?	25-28
**Introduction**	4	One or two paragraphs summarizing why this case is unique (**may include references**)	85-87
**Patient Information**	5a	De-identified patient specific information.	102-48, 167-213, 229-302
	5b	Primary concerns and symptoms of the patient.	102-04, 167-8, 229-32
	5c	Medical, family, and psycho-social history including relevant genetic information	103-8, 169-72, 238-41
	5d	Relevant past interventions with outcomes	127-130, 232-3,
**Clinical Findings**	6	Describe significant physical examination (PE) and important clinical findings.	113-4, 181, 247-51, table 2, line 93
**Timeline**	7	Historical and current information from this episode of care organized as a timeline	table 5 line 425
**Diagnostic Assessment**	8a	Diagnostic testing (such as PE, laboratory testing, imaging, surveys).	116, 181, 233
	8b	Diagnostic challenges (such as access to testing, financial, or cultural)	n.a.
	8c	Diagnosis (including other diagnoses considered)	183, 118, 247
	8d	Prognosis (such as staging in oncology) where applicable	n.a.
**Therapeutic Intervention**	9a	Types of therapeutic intervention (such as pharmacologic, surgical, preventive, self-care)	96 table 3
	9b	Administration of therapeutic intervention (such as dosage, strength, duration)	129-131, 191, 254-5
	9c	Changes in therapeutic intervention (with rationale)	208
**Follow-up and Outcomes**	10a	Clinician and patient-assessed outcomes (if available)	line 99 table 4
	10b	Important follow-up diagnostic and other test results	n.a.
	10c	Intervention adherence and tolerability (How was this assessed?)	266-98
	10d	Adverse and unanticipated events	204
**Discussion**	11a	A scientific discussion of the strengths AND limitations associated with this case report	433-442
	11b	Discussion of the relevant medical literature **with references**.	319-423, 462-514
	11c	The scientific rationale for any conclusions (including assessment of possible causes)	319-423
	11d	The primary "take-away" lessons of this case report (without references) in a one paragraph conclusion	444-9
**Patient Perspective**	12	The patient should share their perspective in one to two paragraphs on the treatment(s) they received	150-164, 215-27, 303-14
**Informed Consent**	13	Did the patient give informed consent? Please provide if requested	Yes ☒ No ☐
